# Abnormally primed CD8 T cells: The Achilles’ heel of CHB

**DOI:** 10.3389/fimmu.2023.1106700

**Published:** 2023-03-01

**Authors:** Xiaoqing Chen, Xue Liu, Yichao Jiang, Ningshao Xia, Chao Liu, Wenxin Luo

**Affiliations:** ^1^ State Key Laboratory of Molecular Vaccinology and Molecular Diagnostics, National Institute of Diagnostics and Vaccine Development in Infectious Diseases, School of Public Health, Xiamen University, Xiamen, Fujian, China; ^2^ Xiang An Biomedicine Laboratory, Xiamen, Fujian, China; ^3^ The Research Unit of Frontier Technology of Structural Vaccinology, Chinese Academy of Medical Sciences, Xiamen, Fujian, China; ^4^ State Key Laboratory of Cellular Stress Biology, School of Pharmaceutical Sciences, Xiamen University, Xiamen, Fujian, China

**Keywords:** HBV, CD8 T cells, immune tolerance, T-cell priming, immune therapy

## Abstract

Chronic hepatitis B virus (HBV) infection continues to be a significant public health challenge, and more than 250 million people around world are infected with HBV. The clearance of HBV with virus-specific CD8 T cells is critical for a functional cure. However, naïve HBV-specific CD8 T cells are heavily hindered during the priming process, and this phenomenon is closely related to abnormal cell and signal interactions in the complex immune microenvironment. Here, we briefly summarize the recent progress in understanding the abnormal priming of HBV-specific CD8 T cells and some corresponding immunotherapies to facilitate their functional recovery, which provides a novel perspective for the design and development of immunotherapy for chronic HBV infection (CHB). Finally, we also highlight the balance between viral clearance and pathological liver injury induced by CD8 T-cell activation that should be carefully considered during drug development.

## 1 Introduction

HBV is known to be a noncytopathic virus exclusively infecting hepatocytes (1). Heparin sulfate proteoglycans (HSPGs) promote HBV Dane particle adherence to hepatocytes (2), and then HBV enters hepatocytes *via* sodium taurocholate cotransporting polypeptide (NTCP) (3). Upon entry into hepatocytes, HBV relaxed circular DNA (rcDNA) is released and translocated to the nucleus, where it is converted to covalently closed circular DNA (cccDNA), which is stable and permanent (4). Hepatic cccDNA is difficult to eradicate once a chronic infection is established. On the other hand, genomic integration of double-stranded linear DNA (dslDNA) may occur, and dlsDNA has recently been shown to be the primary source of circular hepatitis B surface antigen (HBsAg) (5). Adults infected with HBV generally, >95%, develop a self-limited acute-resolving infection (6). In contrast, the establishment of chronicity is more likely when infection occurs during early childhood or perinatally. As estimated, 250 million people worldwide are chronically infected with HBV, resulting in almost 1 million deaths per year from the complications of persistent infection, liver cirrhosis, and hepatocellular carcinoma (7–9).

HBV-specific CD8 T cells with their cytolytic or noncytolytic antiviral function are thought to be the ultimate effectors of HBV clearance (10, 11). According to a previous report, a tissue-resident memory T-cell (Trm)-like phenotype is present in 90% of HBV-specific liver CD8 T cells during chronic infection (12, 13). As memory T cells residing at the location of viral infection, HBV-specific Trm cells exert long-term immune surveillance during viral infection (14). Laura J. Pallett’s research has shown that CD8 Trm cells are preferentially concentrated in partially immune-controlled HBV patients (12). In addition, Cheng’s results proved that longer-term relapse-free survival in HCC patients with chronic HBV infection is associated with the intratumoral infiltration of HBV-specific CD8 Trm cells (15). These pieces of evidence illustrate the important role of liver virus-specific CD8 T cells in the long-term control of HBV infection. However, in CHB patients, HBV viral load and the frequency of intrahepatic CD8 Trm cells are inversely linked (16). This shows that consistent high-load HBV may affect the initiation and differentiation of Trm cells, which leads to a weak adaptive immune response and persistent HBV infection. Understanding how HBV-specific CD8 T-cell tolerance occurs can help people develop effective immune therapies. This article reviews the possible reasons for HBV immune tolerance from the perspective of abnormal HBV-specific CD8 T-cell priming and lists recent studies on immunotherapies for the recovery of virus-specific immune responses, providing a theoretical basis for developing therapeutic drugs for CHB infection.

## 2 Naïve HBV-specific T-cell priming

### 2.1 Location of naïve HBV-specific T-cell priming

**Figure 1:**
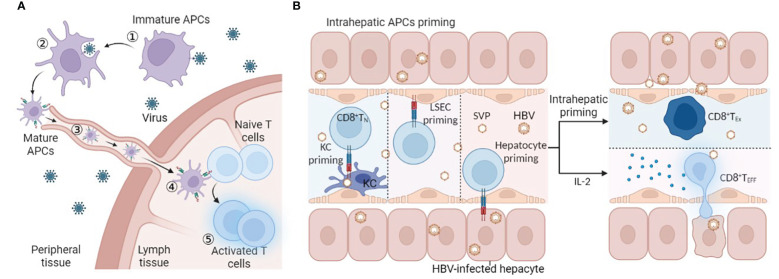
T-cell priming location. **(A)** ①Immature APCs recognize PAMPs in pathogens by PRRs, ② APCs phagocytose and process pathogens, ③ APCs mature and present peptides and migrate into LNs to connect naïve antigen-specific T cells, ④ T-cell priming and activation, and ⑤ homing to the infected site. **(B)** Naïve HBV-specific CD8 T cells circulate through the hepatic sinus and are primed by intrahepatic APCs (KC, LSEC, or hepatocyte). Intrahepatic priming may contribute to the exhaustion of CD8 T cells, and IL-2 can rescue and promote the differentiation of effector CD8 T cells. Abbreviations: APCs antigen-presenting cells, LSEC liver sinusoidal endothelial cell, KC, SVP spherical subviral particle, CD8 T_N_ naïve CD8 T cell, CD8 T_Ex_ exhausted CD8 T cell, CD8 T_Eff_ effector CD8 T cell. This figure was created with *BioRender.com*.

In general, circulating immature antigen presenting cells (APCs) encountering and phagocytosing exogenous pathogens will migrate to T-cell zones of secondary lymphoid organs to activate naïve antigen-specific T cells. The naïve antigen-specific T cells then differentiate into effector cells endowed with the potential to home to infected sites (**Figure 1A**). In HBV infection, the liver is also considered the site of naïve HBV-specific T-cell priming due to the particular structure of the liver: the speed of blood flow in the sinusoid is much slower than that in the periphery (17–19), which allows circulating T cells to recognize MHC-peptide complexes presented by hepatitis APCs, and liver sinusoidal endothelial cells (LSECs) have extensive fenestrae with a diameter up to 200 nm and lack a basement membrane (18), so effector CD8 T cells can project “filopodia-like” protrusions through the fenestrae and contact the underlying APCs in the basolateral layer (20). (**Figure 1B**). However, unlike professional APCs (such as DCs and KCs), nonprofessional APCs (such as hepatocytes) cannot present antigens through MHC II molecules and cannot provide costimulatory signals for T-cell activation (21). Moreover, the liver is an organ of immune tolerance. Therefore, intrahepatic priming is also considered to be responsible for HBV-specific T-cell dysfunction. To date, there is no direct evidence to prove that secondary lymphoid organs or the liver, where there is a high HBV antigen burden, are where HBV-specific T lymphocytes are primed, and the time and location of T-cell priming remain to be further verified.

### 2.2 Barriers of naïve HBV-specific T-cell priming

**Figure 2:**
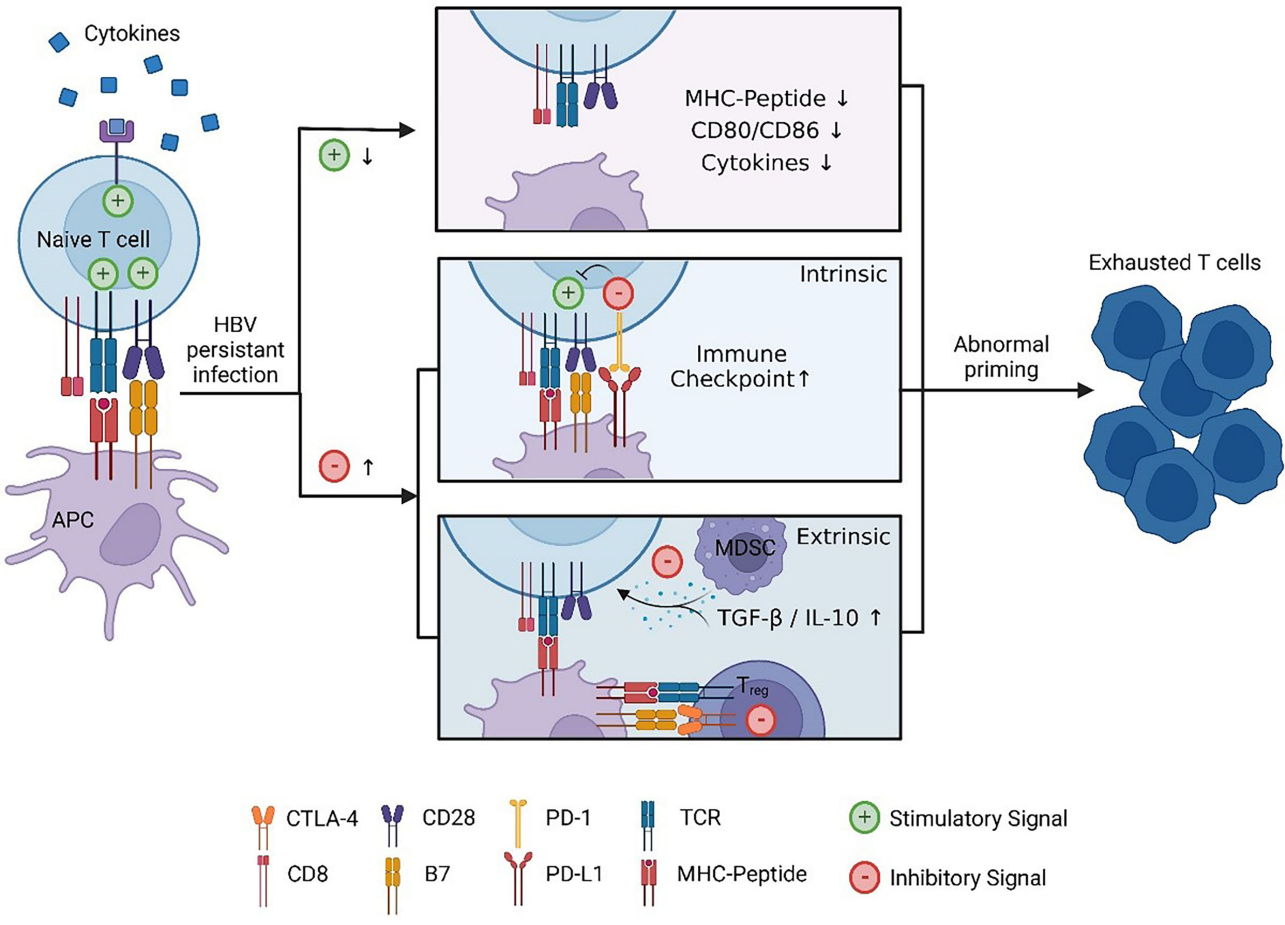
Abnormal HBV-specific CD8 T-cell priming. Due to persistent stimulation with HBV, the stimulus signal is reduced: exhausted APCs cannot provide sufficient signal 1/2 that is manifested in the downregulation of MHC II and CD80/CD86, and cytokine-mediated signal 3 decreases. Moreover, inhibitory molecule-mediated signalling increases: regulatory cells such as MDSCs and Treg cells will secrete anti-inflammatory cytokines, and CTLA-4 expressed in Treg cells will competitively bind CD28 with higher affinity compared to CD80 in APCs. Under these circumstances, T cells cannot differentiate into effector cells, while exhausted T cells lose antiviral function. This figure was created with *BioRender.com*.

Three signals are required in the optimal priming of naïve CD8 T cells. Naïve antigen-specific T cells need T-cell receptor (TCR)-mediated signal 1 as the primary signal to enter the cell cycle and expand (22). Costimulatory molecules expressed in APCs, such as CD80/CD86, bind to CD28 to provide signal 2. Lacking a CD28 signal may cause T-cell anergy in the process of stimulation (23). Moreover, complete activation also requires an appropriate cytokine environment (24). In addition to the stimulatory signal, there is a negative feedback regulation mechanism to prevent excessive activation of T cells. The pathogen can sometimes use this regulatory mechanism to evade immune cell attacks. For chronic hepatitis B virus infection, prolonged exposure to HBV virus or HBsAg/hepatitis B e antigen (HBeAg) may result in the inability of APCs to provide adequate signals 1 and 2 for T-cell priming (25, 26). Combined with the lack of activating cytokines (27) and the interference of inhibitory molecules (28, 29), it may ultimately lead to the dysfunction of HBV-specific CD8 T cells (**Figure 2**).

#### 2.2.1 HBV infection resulted in impaired signal 1 and signal 2, which are required during HBV-specific T-cell priming

HBV is a “stealth” virus that the innate immune system detects only sporadically (30). Generally, pattern recognition receptors (PRRs), such as TLR, NLR, CLR, and RLR, can identify pathogen-associated molecular patterns (PAMPs) and then initiate downstream signalling that activates immune cells (31). In contrast, research proved that HBV could interfere with PRR expression and signalling pathways (32–34), which may result in no response or abnormal activation of innate cells. Moreover, a manifestation of immunological tolerance in APCs can result from prolonged, recurrent exposure to HBV-related antigens. Dendritic cells (DCs) and hepatocytes play crucial roles as APCs in the activation of CD8 T cells specific for HBV. Their ability to recognize HBV and its activation is essential for whether HBV-specific T cells can properly develop into effector cells and memory cells.

The immune function of DCs is abnormal during chronic HBV infection. Many studies have reported that, compared with healthy individuals, DCs from CHB patients showed a decline in capacity for migration, antigen presentation, and cytokine production (35–38). The molecular mechanisms of DC dysfunction remain unknown. However, there is evidence to prove that HBV-related antigens can affect the function of DCs. High-load HBsAg in the periphery exerts long-term inhibitory effects on DCs: Xiaoyan Li’s work has shown that the higher the peripheral HBsAg level in CHB patients, the poorer the maturation status of DCs in PBMCs (25). Moreover, in the PBMCs of patients with HBsAg seroclearance, the frequencies of DCs expressing TLR and CD8 T cells increased, which indicated the negative impact of HBsAg serological load on the function of DCs (39). Tsung-Yu Tsai’s study also proved that HBV could interact with SIGLET-3 through α2-6 connected sialoglycan and then recruit SHP-1/-2 to downregulate the expression of costimulatory molecules and attenuate TLR ligand-induced cytokine secretion (40). Dysfunctional DCs cannot provide sufficient and stable signals for the priming of T cells, which is also one of the reasons for HBV-specific T-cell immune tolerance.

HBV-infected hepatocytes can prime and activate CD8 T cells by presenting antigenic peptides through MHC I. However, it is generally believed that HBV-specific CD8 T cells primed by hepatocytes can be activated and proliferate but are apt to differentiate into exhausted cells instead of functional effector cells (41, 42). Alexandre P. Bénéchet’s work showed that CD8 T-cell dysfunction caused by hepatocellular presentation is different from T-cell fatigue seen in other viral infections and malignancies (42). The unique features of these dysfunctional CD8 T cells are revealed through transcriptomic and chromatin accessibility analyses. The analyses showed that the immune program may prefer tissue protection over antiviral action since genes involved in tissue development, remodelling, cell differentiation, and wound healing are upregulated in this process (42). It is not clear how HBV-infected hepatocyte priming causes T-cell dysfunction. One potential explanation is the downregulation of some molecules necessary for antigen presentation. The spectrum of effector actions of HBV-specific CD8 T cells appears to be determined by the quantity of MHC-peptide molecules (33). Weak TCR signalling can be caused by a 100-fold decrease in MHC I-antigen peptide complexes displayed in HBV-infected hepatocytes (26).

#### 2.2.2 HBV-specific T-cell activation requires activating cytokines as a third signal

In addition to TCR-mediated signal 1 and costimulatory molecules associated with signal 2, the proliferation and activation of the cytotoxic program also require cytokines such as signal 3 (43), such as IL-2, IL-12, and IL-15 (44, 45). However, some HBV components can interfere with the recognition of pathogen sensing pathways during early viral infection, resulting in insufficient proinflammatory and antiviral cytokine secretion (31). These cytokines are essential for HBV-specific T-cell priming, sustained activation, and differentiation to maintain the antiviral response. The results of HBV carrier mice receiving vaccine treatment proved that the loss of serum HBV antigen and DNA was strongly associated with potent and functional HBV–specific CD4 and CD8 T-cell responses and increased production of IL-2, IFN-γ, TNF-α, and IL-12 (46). This phenomenon suggests that the priming and differentiation of HBV-specific CD8 T cells can be remodelled by exogenous supplementation with some cytokines.

Recent studies have determined that intrahepatic APCs such as KCs, hepatocytes, or LSECs can sense IL-2 to promote the priming of T cells. Liver KCs are tissue-resident phagocytes that are capable of maintaining self-renewal (47). They are loaded in liver sinusoids and are crucial for scavenging the immune complex, small particles, and apoptotic cells from blood (48–50). As APCs, KCs can deliver antigens to T cells and promote either effector development or tolerance. Florent Ginhoux’s research team identified KC2 as a subset of KCs in the liver with a specific metabolic function for controlling glucose homeostasis and oxidative stress (51, 52). Their other parallel work further proves that KC2 profiting from IL-2 therapy is essential for reverting T-cell dysfunction and improving the antivirus capacity of HBV-specific T cells (51). IL-2 treatment can also rescue CD8 T cells primed by hepatocytes (42). In mice transferred naïve CD8 TCR transgenic T cells specific for the core protein (Core93 naïve T cells), IL-2c administration facilitated Cor93 T-cell differentiation into cytotoxic effector cells and IFN-γ-producing cells, accumulating in clusters scattered across the liver lobules and exerting substantial antiviral activity (42). IL-2 signalling also controls CD8 T-cell functional programming when liver resident LSECs perform local cross-priming (53). The above research identified that IL-2 can rescue dysfunctional CD8 T cells primed in the liver (**Figure 1B**). In immune-tolerant patients, only when receiving IL-2 treatment can peripheral HBV-specific T cells expand and release IFN-γ after being stimulated with overlapping HBV peptides (42). Effector CD4 T cells can provide IL-2 (53, 54). However, during CHB infection, the cytokine-secretion activity of HBV-specific CD4 T cells is also impaired (27), which may be one of the possible causes of CD8 T-cell malfunction.

In addition to IL-2, IL-12 can boost TCR-mediated signalling, increase antiviral potential, and enhance the effects of coinhibitory blocking (55). Tissue-resident memory CD8 T-cell priming requires IL-12/IL-15 provided by DNGR-1+ DCs, and antibody blockade of these specific signals impairs the priming process (56), which is crucial for the generation of functional HBV-specific liver-resident memory CD8 T cells. Others have shown that IL-12 has the potential to induce IFN-γ-producing T cells and reduce viremia in CHB (57, 58) and HBV-infected animal models (59, 60). Generally, during the priming of naïve HBV-specific CD8 T cells, these cytokines act as the third signal to prevent tolerance induction and cell death (61).

#### 2.2.3 Effects of inhibitory signals on HBV-specific T-cell initiation

As the immune-tolerant organ, there are many inhibitory factors in the liver that affect the antiviral effect of HBV-specific T cells. In some respects, these inhibitory components are a double-edged sword that suppresses the immune response while avoiding tissue damage.

Immune checkpoints are the negative regulatory mechanisms of the adaptive immune response that control the intensity and quality of T-cell activation by interfering with stimulatory signalling. For the PD-1/PD-L1 axis, the B7-1 molecule can interact with PD-L1 (62), and PD-1 can directly recruit phosphatases to interfere with TCR signalling (63). Virus-infected cells or tumour cells upregulate some immune checkpoints to escape immune attack. Most circulating and intrahepatic HBV-specific CD8 T cells in chronic HBV patients express PD-1, which dominates the hierarchy of inhibitory receptors (64, 65). Approximately 90% of vertical transmission leads to chronic infection (66). Recent research revealed that PD-L1 expressed in macrophages of offspring induced by maternal HBeAg suppressed cytotoxic T-cell (CTL) function (67). Moreover, PD-1 on intrahepatic T cells was also upregulated in a mouse model of CHB (68). Therefore, some scholars believe that the activity of virus-specific T cells can partially be restored by blocking immune checkpoint binding to their receptors (69). However, opposing views are also held by others. Bengsch’s results showed that HBV-specific CD8 T cells that reacted to PD-1 blockade exhibited a preference for an intermediate T-cell differentiation stage (65). Recent research has also shown that anti-PD-L1 cannot rescue dysfunctional CD8 T cells that have undergone hepatocellular priming (42). The limited response rate of PD-1/PD-L1 blockade suggested that HBV immune exhaustion results from a complex interaction of inhibition, not a single factor.

Inhibition mediated by regulatory cells, such as T-regulatory cells (Treg cells) or myeloid-derived suppressor cells (MDSCs), represents a cell-extrinsic mechanism of in-trans negative regulation that restrains multiple types of immune cells (70). Treg cells are a group of CD4 T cells that are important in maintaining immune homeostasis. In addition to preventing liver tissue damage, Treg cells can also induce immunological tolerance. The level of circulating Treg cells in CHB patients, especially in patients with more HBV gene copies, is higher than that in healthy people (71, 72). Studies have shown that the activity of HBV-specific CD4 T cells (73) and the proliferation of HBV-specific CD8 T cells (28, 74) can be suppressed by Treg cells. MDSCs are another cell-extrinsic T-cell function regulator under conditions of chronic inflammation (75). Through the expression of Arg1 (76) or PD-1-induced IL-10 (77), MDSCs have been demonstrated to impair the HBV-specific T-cell response during the immune-tolerance phase of chronic infection. In addition, MDSCs from CHB patients could downregulate chemokine receptors *via* TGF-β signalling (29) to affect virus-specific T-cell homing. As anti-inflammatory cytokines, IL-10 and TGF-β perform indispensable roles in virus-related immune evasion. Suppressive monocytes in chronic HBV-infected patients secreted higher levels of IL-10 and TGF-β than those in healthy individuals (78).

In short, the interaction of multiple factors may lead to the abnormal priming of HBV-specific CD8 T cells. Individually or together, impaired TCR signalling, costimulatory signals, regulatory cells and the secretion of proinflammatory or anti-inflammatory cytokines can cause the dysfunction of HBV-specific CD8 T cells. Based on a fuller understanding of the above-described HBV immune tolerance mechanism, numerous immunotherapies have been developed to break HBV adaptive immune tolerance and reinstate the antiviral activity of HBV-specific CD8 T cells.

## 3 Immunotherapy to restore the HBV T-cell response

**Figure 3:**
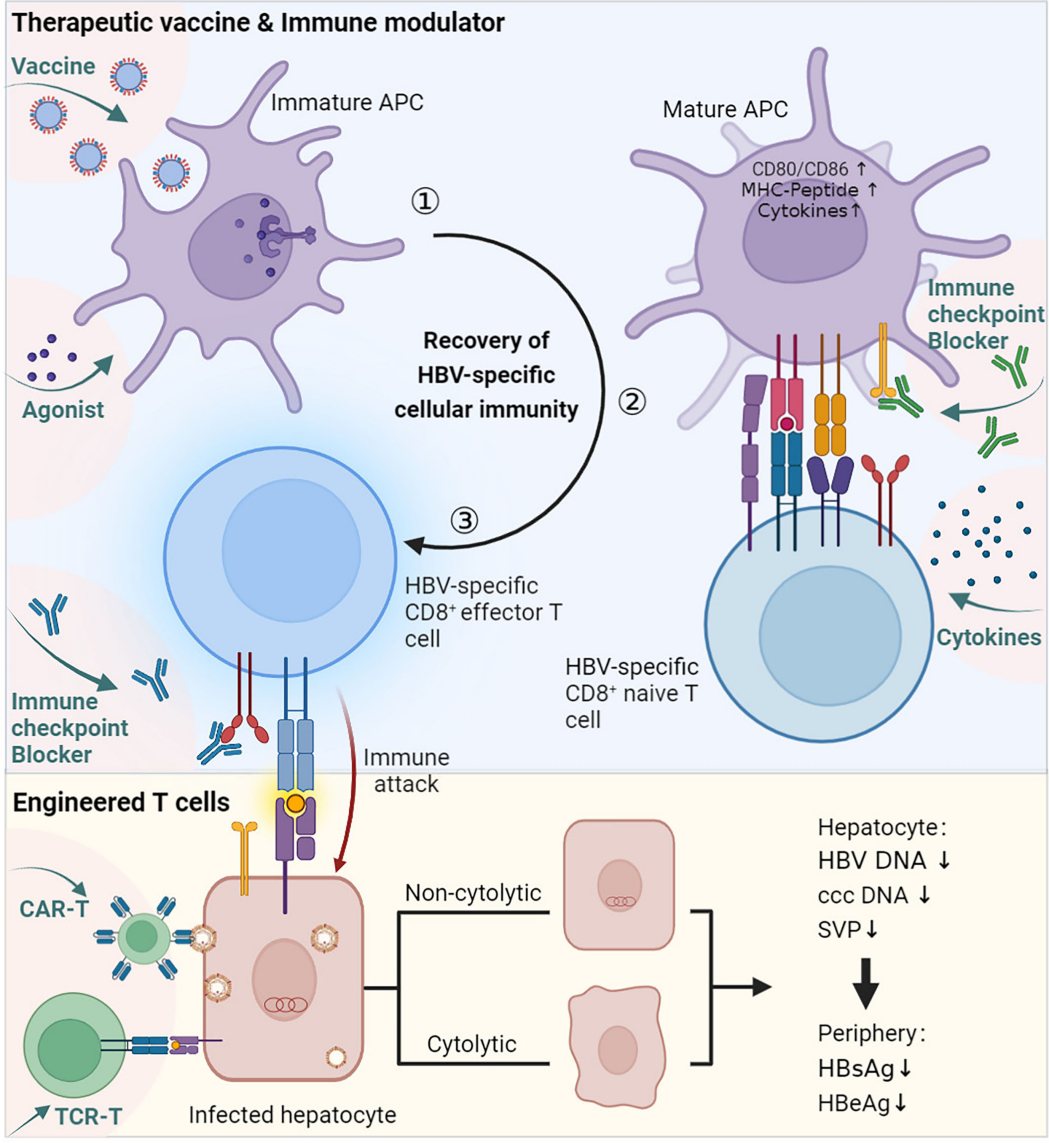
Immune therapies promote the recovery of HBV-specific cellular immunity. ① Vaccine and agonist stimulation. Therapeutic vaccines are generally designed to be taken up by professional APCs and enhance the immunogenicity of virus-associated antigens. The agonist works by promoting the activation of APCs. ② Activated APCs mature and interact with HBV-specific CD8 naïve T cells. During this process, other immune modulators, such as active cytokines, can help activate T cells, and immune checkpoint blockers can prevent inhibitory signalling. ③ Naïve CD8 T cells are stimulated and differentiate into effector cells. Engineered T cells can bypass APC priming and act directly on infected hepatocytes. The immune therapies described above concentrate on the priming progress of HBV-specific CD8 T cells, which mediates cytolytic or noncytolytic immune attacks on infected hepatocytes, ultimately resulting in a decrease in HBV-related viremia. This figure was created with *BioRender.com*.

Currently, treatments approved for CHB are limited to interferon (IFN) or nucleoside or nucleotide analogues (NA). They can control the replication of HBV and limit HBV infection, but both have corresponding disadvantages: they seldom eradicate HBV, so CHB patients have to receive lifelong treatment (79). Therefore, more effective therapies need to be developed against chronic HBV infection. Activating the antivirus immune response allows spontaneous HBV infection resolution, which is considered an essential condition to achieve a functional cure. Cellular immunity is essential for the clearance of infected hepatocytes and virus control. In the above sections, we described the possible causes of the abnormal priming of HBV-specific CD8 T cells, which proved to be important in HBV control. To restore HBV-specific cellular immune responses, researchers have tried to utilize different immune strategies to modulate the immune response, including therapeutic vaccines, immune modulators, immune checkpoint blockade, engineered T cells, and more (**Table 1**, **Figure 3**).

**Table 1:** Immunotherapy for chronic hepatitis B infection.

Name		Description	Mechanism of action & Efficacy	Animal Modal	Research Stage
**Therapeutic Vaccine**	pHBV-vaccine	**rHBVvac+adjuvant**; the recombinant protein vaccine contains HBsAg employed with poly I: C.	The pHBV-vaccine promoted the maturation and antigen presentation ability of DCs, decreased HBsAg and HBV DNA levels in HBV-carrier mice, and protected mice from HBV reinfection (80).	HBV-carrier mice	Preclinical
	HepTcell	**Epitope Vaccine+adjuvant**; contains nine epitope peptides of three different HBV antigens (pol, core, and s) with IC31^®^ (Valneva).	Peptides + IC31 resulted in increased HBV-specific cellular immune responses. No change was noted in antiviral markers above placebo (81).	CHB patients	Clinical Phase II NCT04684914
	GS-4774	**Yeast-based vaccine**; a T-cell vaccine with heat inactivation based on yeast, consists of yeast cells expressing well-conserved HBV protein regions.	GS4774 was well tolerated and enhanced the immune defence against long-term HBV infection (82).	CHB patients	Clinical Phase II NCT01943799
	BRII-179	**VLP vaccine**; an immunotherapy derived from the prophylactic Sci-B-Vac^®^ vaccine based on virus-like particle (VLP) includes the large (L), middle (M), and small (S) envelope proteins.	Patients with chronic hepatitis B responded effectively to BRII-179 alone or in combination with a low dosage of IFN-α, and it was safe and well tolerated (83).	CHB patients under NA treatment	Clinical Phase 1b/IIa NCT04749368
	ChAdOx1 HBV	**ChAd × MVA vaccine**; a novel HBV vaccine using ChAd and MVA viral vectors encoding multiple HBV antigens.	Enhanced the polyfunctional T-cell response and induced the secretion of antivirus cytokines (84).	CHB patients and healthy	Clinical Phase I NCT04297917
	ϵPA-44	**Nanoparticle lipopetide vaccine:** a linear single-chain synthetic long peptide composed of a HLA-A2 restricted CTL epitope, a Th1 cell epitope, a B-cell epitope, and a palmitic acid molecule as adjuvant.	The vaccine was well tolerated in phase 1/2 trials, and induced higher HBeAg seroconversion rate than placebo (85).	CHB patients	Clinical Phase II NCT00869778 Clinical Phase III ChiCTR2100043708
**Immune Modulator**	GS-9620	**TLR 7 agonist**	GS-9620 could induce the production of IFN-α or other cytokines and chemokines and activated ISGs, which induced the clearance of HBV-infected cells (86).	CHB patients	Clinical Phase II NCT02579382
	rIL-2	**Cytokine**	IL-2 treatment can increase the ability of Cor93 T cells to expand and develop into IFN-γ-producing cells with cytolytic potential (42).	HBV-Tg mice and CHB patients(ex-vivo)	Preclinical
	Nivolumab	**Immune Checkpoint Blocker**	Nivolumab’s safety profile was tolerable and has the ability to treat advanced hepatocellular carcinoma, as evidenced by long-lasting objective responses (87).	patients with advanced hepatocellular carcinoma	Clinical Phase I NCT01658878
**Engineering cells**	HBV-TCR RE-T cells	**TCR-T**	TCR-reprogrammed nonlytic T cells have the ability to activate APOBEC3 in hepatoma cells, which prevents viral infection. These hepatotoxic-reduced cells may be used to treat persistent HBV infection (88).	HBV-infected and human hepatocytes transferred mice	Preclinical
	S-CAR grafted T cells	**CAR-T**	The S-CAR-expressing T cells localized to liver and efficiently and rapidly controlled HBV replication, causing only short-term liver damage, and were not impaired or hyperactive by a significant amount of viral antigen (89).	HBV-Tg mice	Preclinical
	LIGHT-DC	**Engineered DC**	LIGHT-modified DCs could induce stronger antiviral immunity by upregulating the expression of costimulatory molecules the secretion of cytokines IL-12 and IFN-γ, resulting in the recovery of HBV-specific CD8 T-cell response and the decrease of serous HBsAg and HBV DNA in HBV transgenic mice (90).	HBV-Tg mice	Preclinical
**Combination treatment**	GS-4774 + Ninolumab	vaccine + PD-1 blocker	Checkpoint blockage was well tolerated in virally suppressed HBeAg-negative patients, and it resulted in HBsAg reduction in the majority of patients and durable HBsAg loss in one patient (91).	CHB patients	Clinical Phase Ib NCT02174276
	GS-4774+TDF	vaccine + NA	Therapy that immune TDF with GS-4774 on CHB patients could enhance the production of *IFNG*, *TNF*, and *IL2* by CD8 T cells exposed to antigenic peptides, but had minimal impact on CD4 T cells (92).	CHB patients	Clinical Phase II NCT02174276
	Peg-IFN-α-2b + IL-2	IFN + cytokine	Following sequential therapy with IL-2, the numbers of Tregs and PD-1 expressing cells decreased and rescued practical immune function involving STAT1 activation, increasing the frequency and function of HBV-specific CD8 T cells (93).	CHB patients with or without IFN-α therapy	Clinical Phase II ChiCTR-INR-17010841

### 3.1 Therapeutic vaccine

Therapeutic vaccination aims to stimulate an immune response against persistent HBV infection. Various therapeutic vaccine strategies for stimulating cellular responses have been explored. The Chimigen^®^ HBV immunotherapeutic vaccine was designed to target DCs that trigger both humorall and cellular immune responses in sheep (94). An rHBVvac therapeutic vaccine based on poly I: C successfully restored exhausted CD8 T cells and partially destroyed the immune tolerance developed in HBV-carrier mice (80). HepTcell is a fluropeptide vaccine tested in phase 2 clinical research and was demonstrated to activate CD4 and CD8 T cells in HBV carriers regardless of HLA background. In a phase 1 clinical trial of HepTcell, all doses of HepTcell were well tolerated, and a group of peptides combined with IC31 adjuvant showed potent HBV-specific cellular immune responses against HBV antigens (81). ϵPA-44 is another peptide vaccine containing 3 different epitopes (**Table 1**) and a palmitic acid molecule as an adjuvant. Among HLA-A2-positive CHB patients, sustained treatment with ϵPA-44 can improve the HBeAg seroconversion rate more than placebo. However, according to the results in peripheral blood, the presence of functional heterologous T-cell subpopulations within the patients’ preexisting immunity is still a key factor in the recovery of the CTL immune response (85). A heat-inactivated T-cell vaccine made of yeast called GS-4774 was designed to enhance the immune system’s defences against persistent HBV infection. In clinical phase II, GS-4774 was well tolerated but unable to decrease serum HBsAg in CHB patients (82).

Moreover, multiple antigen-displayed vaccines have been developed to enhance immunogenicity and antigen-specific immune responses. BRII-179 (VBI-2601) is a novel protein-based recombinant vaccine comprising HBV surface envelope proteins (pre-S1, pre-S2, and S). A randomized phase Ib/IIa study was developed to assess the safety, antiviral activity, and immunogenicity of BRII-179 with the coadjuvant IFN-α. BRII-179 showed an excellent safety profile in CHB patients receiving NA treatment and evoked HBV-specific B and T-cell immune responses (83). Another novel vaccine, ChAdOx1 HBV, encoded several HBV antigens using modified vaccinia Ankara (MVA) and chimpanzee adenovirus (ChAd) viral vectors. The amplitude and breadth of the vaccine-induced polyfunctional T-cell response could be further improved by ChAd-prime followed by MVA-boost vaccination, and the secretion of TNF-α, IFN-γ, and IL-2 could also be induced (84). This work has just completed a phase 1 clinical evaluation.

### 3.2 Immune modulators

#### 3.2.1 Agonist

HBV therapeutic vaccines have a good immune activation effect when used in combination with adjuvants (80, 95–97), indicating that the stimulation of innate immunity is crucial for the recovery of the HBV-specific immune response. Agonists can help CHB patients activate their innate immunity (98). TLR agonists can activate TLR-mediated pathways, suppressing virus replication and immune activation *in vitro* and *in vivo (* 99). Several TLR agonists are currently in the clinical stage (96, 100, 101). A TLR7 agonist (GS-9620) could activate interferon-stimulated genes (ISGs) and induce the production of IFN-α, as well as other cytokines and chemokines, which led to the clearance of HBV-infected cells (86). In phase 2 research, GS-9620 was well tolerated and safe in CHB patients, transcription of ISGs and serum cytokines was detected, and no clinically significant HBsAg decline was observed (102). Coincidentally, recent research has shown that HBV can disturb host cGAS DNA sensing to enhance the replication of HBV (103). Therefore, Andrea Brazdova’s team used PBMCs and nonparenchymal liver cells from healthy human donors and HBV-persistent or control mice to prove that STING agonists can induce the secretion of antiviral cytokines and trigger hepatic T-cell activation *in vitro (* 104).

#### 3.2.2 Cytokines

In addition to agonists, some cytokines conducive to T-cell activation have also been used to treat chronic hepatitis B virus infection. As mentioned above, IL-2 treatment can rescue HBV-specific CD8 T cells primed intrahepatically. In addition, experimental results derived from persistently HBV-infected mice show that gene and cellular therapies based on IL-21 may be candidates for treating chronic HBV infection since they can activate and boost CD8 T-cell infiltration in the liver (105, 106).

#### 3.2.3 Immune checkpoint blockade

During the recovery of the adaptive immune response, sustained HBV-associated antigen stimulation may induce the upregulation of immune checkpoints, which usually leads to T-cell dysfunction (107). Therefore, immune checkpoint inhibitors are effective in several malignancies (79). Regarding T-cell exhaustion in CHB patients, checkpoint inhibition is also considered to have some therapeutic potential. In CHB patients, antibodies that disrupt the interaction between PD-1 and PD-L1 can partially restore the faulty T- and B-cell response to HBV (108, 109). Two anti-PD-1 antibodies are currently under clinical evaluation (Nivolumab in phase I (87); Cemiplimab, REGN2810 in phase I/II) (79). Nivolumab had a tolerable safety profile and an objective response rate of 20% in patients treated with nivolumab 3 mg/kg in the dose-expansion phase of an open-label, noncomparative, phase 1/2 dose escalation and expansion trial in patients with advanced hepatocellular carcinoma with or without HBV infection (87). This clinical result suggests that PD-1/PD-L1 blockers have some therapeutic benefit, but the response rate is limited.

### 3.3 Engineering cells

In addition to improving immunotherapeutic methods, developing techniques to engineer antiviral immune cells holds promise (9). Although the complicated T-cell dysfunction system has no clear explanation, engineered cells can bypass this hurdle to achieve viral elimination. A variety of approaches to engineering immune cells have been developed, such as CAR-T cells, TCR-T cells, and engineered DCs. TCR-reprogrammed T cells and HBV-TCR RE-T cells can perform antiviral functions in a nonlytic way and successfully control viral infection (88). Another study proved that CD8 T cells engineered with chimeric antigen receptors containing a variable domain of an anti-HBsAg-specific antibody to target HBV-infected hepatocytes could rapidly and efficiently control HBV replication with transient liver damage in mice (89). W. Jiang’s research team modified bone marrow-derived dendritic cells (BMDCs) with an adenovirus vector expressing mouse LIGHT to enhance the production of costimulatory molecules and cytokines, both of which are required for DC maturation and CTL activation (90).

### 3.4 Combination treatment

Currently, there are many immunotherapies for chronic HBV infection; however, it is not easy to achieve a functional cure by monotherapy due to the complexity of the HBV immune tolerance mechanism. Therefore, combination therapy is a sensible approach. A good combination therapy strategy can combine the advantages of two or more immunotherapies to achieve the goal more safely and effectively. A variety of combination therapy strategies are currently under clinical evaluation, including vaccine and immune checkpoint blockers (91), vaccines and NAs, IFNs and cytokines. In a phase 2 study, patients given tenofovir disoproxil fumarate (TDF) with GS-4774 exhibited increased production of cytokines. However, the combination failed to decrease the levels of HBsAg (92). In a clinical trial of IL-2 therapy, the majority of CHB patients finished their course of IFN- and IL-2 therapy, and those who were receiving entecavir (ETV) continuously had a significant decrease in HBeAg and a rise in the percentage of HBV-specific CD107a+/IFN-+ CD8 T cells. One patient had HBsAg loss, while five of them showed HBeAg loss (93). This clinical trial suggested the efficacy of IL-2 therapy followed by IFN-α (93). The safety and therapeutic efficacy of combination treatments have yet to be further evaluated.

### 3.5 Others

In recent years, some new immunotherapies have appeared, and attention has been drawn to the contribution of intracellular metabolism to HBV-specific T-cell dysregulation. -HBV-specific CD8 T cells from patients substantially downregulate various cellular processes centred on extensive mitochondrial alterations (110). Furthermore, polyphenol treatment was shown to simultaneously target multiple altered intracellular pathways with a combination of mitochondria-targeted antioxidants and natural polyphenols and improve the antiviral function of T cells (111).

In conclusion, immune tolerance caused by ineffective innate and adaptive immune responses is challenging to recover spontaneously in the body. Thus, artificially modulating the immune response through various approaches can potentially restore the HBV-specific cellular immune response. Therapeutic vaccination seems to be a potential treatment strategy for chronic HBV infection. However, many factors need to be taken into account, such as epitope efficacy, HLA restriction, immunogenicity, and vaccination strategy. Moreover, the therapeutic effect of therapeutic vaccines largely depends on the agonistic effect of adjuvants. In addition, immune modulators have the potential to reshape the immune environment. However, efficacy and systemic toxicity need to be balanced carefully by optimizing dosing and targeting. In summary, these immunotherapies may be able to allow naïve HBV-specific CD8 T cells to be successfully primed and activated, differentiate, and perform antiviral effector functions. Finally, the clinical effectiveness and safety of different therapeutic vaccines remain to be further tested.

## 4 Discussion and conclusion

HBV-specific CD8 T cells are essential for HBV clearance from HBV-infected animal models or patients. In CHB patients, the frequency and efficiency of HBsAg-specific T cells are inferior to those in patients with acute hepatitis B (AHB) virus infection. The reasons include the central and peripheral tolerance caused by repeat exposure to HBV-related antigens. There is no direct evidence for central tolerance in CHB patients to prove thymic deletion of T-cell clones. Recently, Fang et al.’s work based on mice certified that high-load HBsAg promotes polarization of monocytic MDSCs (mMDSCs) from monocytes (112) and induces the expression of chemokine receptor CCR9, which leads mMDSCs to migrate to the thymus and promote the death of HBsAg-specific CD8 thymocytes (113). These results partly explained why infection with HBV in early childhood causes chronic infection but not in adults. According to the current results in mice, the correlation between central immune tolerance and T-cell epitopes cannot be ruled out because the results are based on HLA-A2-restricted epitope-specific T cells (113). Nevertheless, the conclusions about the central immune tolerance of HBV-specific T cells are controversial because T-cell clones specific for HBV remain detectable in fully developed HBV transgenic mice exposed to viral antigens at birth, although most of them are dysfunctional (114, 115). This suggests that other tolerance mechanisms exist. In chronic HBV infection, the heavy load of HBV-related antigens and the repetitive activation of these antigens significantly impair immune cell function. Most of these cells are in a hyporesponsive state.

In the past four decades of HBV research, there have been various attempts to break immune tolerance. In our previous studies, the therapeutic antibody E6F6 targeting a unique epitope of HBsAg (116) and its epitope vaccine CR-T3-SEQ13 (117) showed potential humorall immune recovery capabilities. For cellular immunity, it has been believed that reducing the level of HBV-related antigens in the periphery or in the liver might awaken CD8 T-cell immunity and achieve a functional cure (118, 119). Nevertheless, recent studies found that inadequate HBV-specific T-cell responses were more closely connected to the age of the patients, hepatitis B core-related antigen (HBcrAg), and the duration of exposure to HBsAg than the quantity of HBsAg (120–122). HBsAg clearance may be a necessary but insufficient condition for immune system recovery. Coincidentally, antiviral strategies to control the level of HBV antigen in the liver or periphery are insufficient to drive substantial immune control of prolonged HBV infection (118). Therefore, exogenous immune stimulation or immune checkpoint blockers are necessary to aid in the recovery of the HBV-specific immune response. For the complicated immune mechanism of HBV infection, it is necessary to comprehensively consider various immune tolerance factors, so combination therapy to combine the benefits of multiple immunotherapies is a sensible way to break the immune tolerance of chronic HBV infection. For example, combining the clearance of high-load circulating HBsAg with immune stimulation and therapeutic vaccines combined with immune agonists may be a potential treatment strategy. More work should be done to reveal which method is more effective.

HBV-specific CD8 T cells mediate viral clearance by directly acting on virus-infected hepatocytes through cytolytic and noncytolytic mechanisms (123). Therefore, it is worth noting that virus-specific CD8 T cells are associated with viral control and tissue damage. Treatment is typically not advised for chronic HBV patients during the immunological tolerant period since HBV is a noncytolytic virus (124). The reason is that once the immune system is activated, the spatiotemporal coexistence of hepatocyte injury, apoptosis, and regeneration may lead to abnormal repair mechanisms and random genetic damage, accelerating hepatic disease progression (10, 125). Therefore, many factors need to be considered during the treatment of CHB infection, such as the ratio of noncytolytic and cytolytic effector function of virus-specific CD8 T cells and whether there is a bystander effect on the cytolytic effect. Accurately detecting intrahepatic infected hepatocytes is a precursor to judging whether immune treatments will cause severe liver damage or functional cure. Moreover, patients with different liver environments may react differently to the same immunological stimulant. Thus, whether patients can withstand intrahepatic immunity activation brought by exogenous stimulation still needs a more accurate assessment of the intrahepatic environment (98).

In conclusion, the immunological response against HBV infection requires HBV-specific CD8 T cells. However, some studies indicate that naïve HBV-specific T cells do not differentiate into effector cells as a result of improper priming, ultimately leading to cellular exhaustion. As we described above, the environment of the priming location, the stimulation signal provided by APCs, CD4 helper cells, or other innate immune cells, and inhibitory signals also contribute to the abnormal priming of naïve CD8 T cells. Therefore, the recovery of HBV-specific T-cell immune activity is a promising treatment path to obtain a functional cure. Therefore, single-drug and multidrug combination therapies are expected to achieve a functional cure for HBV. In the future, the molecular mechanism of T-cell tolerance remains to be further studied. Understanding these mechanisms can help us design new, more reasonable, and effective HBV treatment strategies.

## Author contributions

WL, CL and NX conceived the structure of the paper. XC, XL and YJ wrote the manuscript and created figures. All authors contributed to the article and approved the submitted version.
